# The Impact of Quality of Life on the Health of Older People from a Multidimensional Perspective

**DOI:** 10.1155/2018/4086294

**Published:** 2018-05-16

**Authors:** Luis Miguel Rondón García, Jose Manuel Ramírez Navarrro

**Affiliations:** ^1^Faculty of Social Work, University of Granada, Granada, Spain; ^2^Faculty of Social Studies, University of Málaga, Málaga, Spain

## Abstract

**Background:**

This research analyzes the impact of quality of life as a metavariable that conditions the health and social welfare of the elderly. The sample of this study is composed of 500 people, randomly selected from the major day centers for the elderly in the province of Granada (Spain).

**Method:**

For the inferential analysis, we used the CUBRECAVI questionnaire, which is a multidimensional scale of health and quality of life, along with the Katz and García measure questionnaires, which are also applied to quality of life. Through the technique of the interview, we have distributed the participants into two groups: experimental and control.

**Results and Conclusions:**

Once the tests have been completed, we have concluded from the meta-analysis and validation tests that the participants have a good perception of their quality of life, considering health, leisure, environmental quality, functional capacity, level of satisfaction, social support, social networks, and positive social interactions as the determinants of their well-being, although social contact reduces as the age advances. We conclude that multidimensional evaluation is an effective tool to evaluate the quality of life and the objective and subjective health of the elderly. These variables can be related to the improvement of health and well-being.

## 1. Introduction

The World Health Organization [1] defined quality of life as an “individual perception of his or her living situation, understood in a cultural context, value system and in relation to the objectives, expectations and standards of a given society” (p. 2). From this perspective, health-related quality of life includes areas such as physical health, psychological state, level of independence of the person, personal relationships, beliefs in a particular context or the natural environment, social support, and perceived social support [2–6].

The different discursive approaches have recognized the importance of implementing health measures from a multidimensional perspective. This means that while analyzing the quality of life, factors such as the various social conditions and social, cultural, and psychological networks that exist within the different study groups should be considered [7–13]. We add social networks, which are a fundamental element to understand this analysis, as they are an important vector of the quality of life.

However, for a general approach to the state of health from the quality of life perspective, it is necessary to consider more precise questions, and it is, therefore, important to distinguish health from life satisfaction, which involves complacency with the life of the present and past experiences. In this sense, many gerontologists claim that older people who successfully age are those who feel happy and satisfied with their past and present and enjoy positive social relationships and contacts. This concept also refers to a subjective dimension of welfare, to an adequate capacity to adapt to, accept, and recognize the environment, in order to have a better perception of health and welfare. It is about explaining how people experience their lives, their cognitive assessment, their emotional reactions, and their adaptation to life.

As we have already mentioned, quality of life and life satisfaction are different concepts, but at the same time, they are complementary. Life satisfaction represents an indicator of quality of life, a specific part of it, since it focuses on moral, cognitive, and affective aspects, on the tasks carried out independently, and on satisfaction with social support received and, in general, is related to life expectancy [14, 15]. On the contrary, quality of life is more closely linked to factors strictly related to health [16]. To put it more accurately and pragmatically, many older people relate quality of life to the ability to be independent in their daily activities. This is the reason why it is so important to take into account the improving self-esteem, satisfaction with functioning, a sense of independence in daily life tasks, and a sense of participation, which are important components of the whole structure that makes up the standards of quality of life of the elderly.

From this perspective, it is common in professional practice to measure the quality of life according to signs of satisfactory living. More recently, the cultural context, the meaning of life for a person from a quality of life perspective, has been introduced. This shows that quality of life, in addition to being multidimensional, must take into account the person's life experience and how they feel and interpret their life in relation to other people involved. This idea becomes more relevant in people who suffer from dementia, who need to foster their empowerment, so that they can express how they feel and their needs, when more specific stimulation is required for them.

It is worth mentioning that, in recent decades, research is related to health, quality of life, and gerontology as linked subjective health with psychological welfare beyond the absence of disease [17–19]. The Gwozdz and Sousa-Poza study [20] shows that happy people can live longer, and this idea envisions interesting research prospects for the future. Previous research has already highlighted the relationship between the age evolution and happiness in the elderly [21–24]. In this sense, and in line with our main research objective, professionals must look beyond the aspects related to the health of older people, that is, how to restore psychological and social crisis, as this leads to acceptance of the ageing process. Results of White's study assessed “life experiences, describing personality, past traumas, social support, and level of activity, along with physical and psychological health, influencing levels of happiness and satisfaction in the elderly” (p. 54). These findings explain the need to review the variables involved in success or satisfaction with life, such as psychological and physical health, level of activity, and social support. The combination of these factors can give a sense of usefulness and a positive feeling in the review or balance of life in the elderly.

Other specific factors should also be considered when analyzing life satisfaction in people with reduced capacity or dependency. In these specific cases, social, physical, and economic aspects interact with each other as a combination that determines the optimal quality of life [25]. Especially the feeling of loneliness, the degree of self-care capacity, the feeling of concern, the scarcity of financial resources to manage personal independence, and the basic activities of daily life in relation to real needs are the factors that condition health [26].

To finish with, we conclude that no clear consensus exists over the definition of quality of life in elderly to develop the concept based on all its extent for further evaluation from a psychological, cultural, or social point of view. In fact, instead, there is a wide variety of terminology that includes a satisfying life, subjective and psychological welfare, personal development, and other elements that many authors consider synonymous with a satisfying life and welfare. These authors found some approaches to the three dimensions of the concept proposed by Borthwick-Duffy [27] by adding a fourth dimension that seems key to our study: the satisfaction experienced by people in their living conditions, the combination of subjective and objective elements along with the sensations they experience, according to their scale of values, personal expectations, and aspirations held by all elderly people.

From the above analysis, it is evident that the quality of life, in addition to being multidimensional, must take into account the person's life experience, how they feel, and how they interpret their lives [28]. These factors, along with good habits, social support, and relationships, significantly affect health.

Finally, and as a synthesis, the ability to evaluate and learn about these elements allows us to manage health and disease and to identify those factors that can affect. It also improves the quality of life of older people from an integral point of view, that is, from a medical, psychological, and social perspective. It is a multifaceted, interdisciplinary, holistic approach, focused on the person searching for new support strategies for the care and improvement of the health of the elderly, from an interdisciplinary point of view. This approach is beginning to be implemented in clinical practice in some projects of care for dependent and independent elderly people. In summary, the main objective of this research is to analyze the quality of life and the perception of health of the elderly from a multidimensional perspective. The following objectives are pursued:

- To assess health in general, from a subjective and objective point of view
- To assess functional, recreational, and leisure skills
- To analyze life satisfaction, taking into account the social contacts and networks, and other social variables
- To determine the relationship between the variables described above and how they complement each other

## 2. Materials and Methods

According to data from the National Institute of Statistics [29] for the year 2017, the total population over 65 years of age in Granada in 2017 is 47,257 (20.48%). Based on these data, the sample group is made up of 510 people, selected at random from the municipal population census, from five social centers for the elderly in the province of Granada (Spain). This type of random sampling is suitable for this type of research, since all people in the universe have the same probability of being chosen, discarding those who, due to their dementia or disability, could not collaborate in the survey.

The selection of participants was coordinated with the help of a technical team. Each of the interviewers chose a center to conduct the random questionnaires, selected from the available list of the Health and Social Services. The surveys have been thorough and were conducted from January to May 2017. In order to improve effectiveness, four months elapsed between the first evaluation and second evaluation, with the time needed for a rigorous analysis of the results obtained in the two groups studied. Before starting, a pilot study was carried out to adapt the items or questions to the characteristics of this sector of the population. In both cases, the sample had sociodemographic and health characteristics similar to those of social center users, with representative quotas by sex and age, and an age range of 60 to 85 years for both genders. The experimental group was composed of 250 people, and the control group was composed of 260 people.

The applied research design has been a longitudinal panel design. In the first part of the empirical research, the statistical tool SPSS version 22 was used for the inferential analysis, based on the data obtained from the validated questionnaire CUBRECAVI [30], which is a reliable and valid scale for multidimensional measurement of health and quality of life. It is a validated questionnaire that has the psychometric guarantees of reliability and internal consistency and is very appropriate to our research objectives. It is easy to complete, of short duration (approximately 20 minutes), and measures the following areas related to quality of life: health (subjective, objective, and psychic), social integration, functional capacities, activity and leisure, environmental quality, life satisfaction, education, income, and social and health services. By means of individual and group interviews, we have arranged the participants into two groups: experimental and control. In order to analyze the reliability of the CUBRECAVI scale, the Cronbach alpha index was applied to the main variables that made up the questionnaire: health, integration, activity and leisure, functional capacities, and environmental quality. Subsequently, a regression analysis was conducted, based on the responses to the CUBRECAVI questionnaire, in the experimental and control groups. This was followed by a comprehensive statistical analysis of the most significant variables related to social relations, health, and quality of life. This analysis involved social contacts, frequency, social networking, and cohabitation with family members. The questionnaires included sociodemographic data, social support OARS, daily activities, health-related quality of life scales (QL-Index), and the Garcia scale [31, 32]. These scales were designed to assess the frequency and degree of satisfaction with the social contact received from different sources in relation to quality of life. This study also tested the reliability of the questionnaire, its criteria, and its structural validity, already demonstrated by the authors who validated the tool. The correlative analysis showed significant positive associations between the scores obtained and measures of the explanatory or independent variables, as explained below. Finally, a multivariate descriptive analysis was carried out with the sample studied.

For the analysis of inferential statistics, descriptive results and sociodemographic variables were initially analyzed. Later, we studied both the magnitude and the significance of the association between the dependent variables and each of the independent variables (Mantel–Haenszel chi square) in both the experimental and control groups. The magnitude of the associations is expressed with odds ratios and the differences in percentages, as well as their reliable intervals. In contrast to the hypotheses of the quantitative variables, techniques such as Student's and analysis of variance were used if the necessary conditions were met. In order to identify independent risk factors, multivariate analysis techniques and nonconditional logistic regression with binary dependent variables and Cox regression were performed. All these, as indicated, are performed with the SPSS statistical tool.

## 3. Results

In the descriptive results of the sociodemographic variables, we found that the percentage of men is slightly higher than that of women, which represents 43.6%. In terms of age, all of them are older adults aged between 60 and 70, with 43% of the sample being between 60 and 70 years old, compared to 57% between 70 and 87 years old. The socioeconomic level of the participants is medium low, with an average income ranging from 500 to 600€ per month, although they are within the national average. No significant differences have been found in gender dimensions or educational levels, although age is significant. Hence, the older the person, the greater the deterioration in the quality of life.

As soon as the sociodemographic data were identified, we examined the specific components of quality of life which are most relevant for the older participants in the study of both groups. First, in Table 1, we began to analyze the internal consistency of each of the scales indicated by CUBRECAVI to measure the reliability of this tool. For this purpose, the Cronbach alpha index has been calculated for the subvariables: health, social integration, leisure and activities, functional capacities, and environmental quality. As the scales have only one item, an index of the five scales indicated above has been calculated.

The previous results indicate that the scale used in our research (CUBRECAVI) allows us to predict a subjective criterion of quality of life, that is, whether the subject is satisfied with life. As explained at the beginning of this document, satisfaction with life is related to quality of life, although specific aspects of each of these concepts are measured. According to the data in Table 1, the internal consistency indices are moderate (between 0.81 and 0.61) in all the subvariables (health, leisure and activities, environmental quality, and functional capacities), with the exception of the social integration that it is low (0.22).

Once the reliability of the scale has been verified, in Table 2, we proceed to apply the regression analysis with the five variables indicated by CUBRECAVI and analyzed in our questionnaires already indicated in the table.

In the multiple regression process, the aim is to describe the impact of the quantitative factors applied on the CUBRECAVI scale on the dependent variable proposed in our study on quality of life, which in turn is related to the health of older people, which, as we have explained in the initial introduction, usually have multiple causes. In this specific case, health and quality of life phenomena relate to several independent factors or variables that influence a priori, such as health, functional capacities, leisure and activities, environmental quality, and income. For this reason, multivariate analysis analyzes the five variables simultaneously, relating them to the impact on the quality of life of the elderly, in order to know how they can affect. It is a question of finding out from a statistical research whether they are in any way related to each other, so it is possible that they can be mathematically related. In order to achieve this purpose, we calculate the regression line in Table 2, where we represent the results of this parametric test with the two groups studied:

Once the regression analysis was carried out, as shown in Table 2, which differentiates the results of the experimental and control groups, we concluded that health, leisure, activity, and environmental quality are the significant variables with the greatest influence on older people in both groups. In detail, in the experimental group, four of the five variables established by CUBRECAVI have been validated as determinants, with the exception of income. These values represent 25% of the total variance in the experimental group. In the control group, the same thing happens as in the previous case, and only the income variable is not significant, where the variance represents 31% of the total.

The results of the two groups have in common the importance given to leisure, spare time, health, and the influence of these factors on the quality of life. However, the participants in the control group attach more importance to functional skills and leisure activities. In contrast, in the experimental group, they value environmental conditions more. These differences, nevertheless, are minimal.

Therefore, we understand that the elderly consider transcendental, for their lives, having the capacity to carry out the basic activities of daily life, but also having sufficient physical capacities to allow them access to leisure and spare time, in an suitable environmental context. In simple terms, this is interpreted as the need to be able to carry out their personal autonomy and community participation in the environment, or the natural environment, close to their reference nuclei, taking into account the limitations of age. After all, being well, being able to do the basics for oneself, and enjoying spare time are priority aspects in this stage of life because they make it possible to feel more empowered and better. For obvious reasons, the connotations of these elements are direct with the state of health because they influence the fact of feeling better and, consequently, of being better, of the perception one has of one's health and quality of life.

To complement the results of the previous analysis obtained in the factors determined by the scale CUBRECAVI, a new element has been introduced, which in our opinion may be of interest for the purpose of this study. We are referring to the effects that social contacts and social relations have on the quality of life of the elderly and, of course, also on their health situation. To this end, we have taken into account what has been published in the latest research and articles in the area of geriatrics and gerontology that places contact, its duration, frequency, and interactions as a crucial axis in the health and illness binomial. Thus, in Table 3, we show the multivariate analysis of nonconditional logistic regression in the same subjects, with both models, in relation to the independent variable called social contact. Three specific aspects or dimensions are calculated: the association between the duration of visits, weekly contacts, and cohabitation as explanatory subvariables. The data are intended to determine whether or not the probability of these variables occurring will influence the quality of life and health of the elderly and whether they are a complement to the variables studied in the table above. The strength of association is expressed in odds ratios.

If we compare the variables set out in Table 3 in the results of both groups, we find some differences in the scores obtained. First, in the control group, we find negative associations in the coexistence subvariable, when contact with children occurs (HR 0.99), and positive association when it is with other family members (HR 1.28). HR stands for the hazard ratio. Also, as reflected in the odds ratio data, they are negative when the number of visits per week is increased. Specifically, the association is more negative when the visits are more frequent than two per week (HR 0.82) but also when there are no visits at all (HR 0.79). On the contrary, 1 or 2 contacts with people per week are positive for this group (HO 1.08). We can conclude from these data that the ideal is to have visitors, but not too many, given that elderly value quality more than quantity.

Secondly, in the experimental group, there is a positive association with the quality of life and health of the elderly, in the variables called coexistence and contacts, but also with divergences from the previous group. In the case of contacts, both the length of time they last and the frequency or number of times they occur influence this model. That is to say, the longer and more frequent the visits, the better the association or dependency. Hence, we can consider that the experimental group does value the frequency and number of social interactions and not so much their quality as the other group does.

Specifically, by analyzing each of the three dimensions of contact in experimental and control duality, the differences in scores are minimal. It is interesting to observe results in common, in the variable related to coexistence. In particular, this relationship is more positive, with second-degree relatives and other persons outside the primary group, than with those closest to them, such as the spouse or children. That is to say, other members than the direct family provide a more positive relationship in the elderly of both groups, with an even greater incidence in the control group. We can conclude, then, that in this study, the incidence of the contact subvariable is clearly a determinant of the quality of life of the experimental group with an HR between 1.04 and 1.40, and a 95% confidence Interval, compared to the control group with an HR between 0.79 and 1.08. In any case, in both groups, it is closer to the value 1 when the contacts range from 1 to 2 per week. This means that older people who have social contacts are more likely to improve their quality of life when they have weekly contacts with others, regardless of kinship.

As for the variable related to the contacts made with different people during the week, although they are valued as important in general, it seems that they are more positive when they are of a medium frequency but not intense. The ideal is between 2 and 4 contacts per week in the experimental group (HR 1.401). In this respect, the two groups do not agree on this point, and the number of contacts per week is more important for the experimental group. The common point, or coincidence, occurs when considering two average contacts per week as the average of positive contacts for quality of life.

We insist once again that social relationships depend on several factors as determinants of quality of life. For this reason, in addition to coexistence and contacts, the length of time these contacts last is also important. According to these results, as for the number of visits, the opposite occurs when the visits decrease to none per week, which is a negative association in the experimental group. When they increase, that is, between 2 and 5 visits per week (control group HR 1.024), it is considered a positive element in the perception of quality of life. In the control group, the scores are slightly lower, peaking at 1.182 in 1 to 2 visits per week. Consequently, the higher the number of visits, the better the quality of life. That is, having visitors or contacts is an important predictor as well, with a clear positive association with the dependent variables.

## 4. Discussion

Data from multivariate analysis show that the determinants indicated in the variables analyzed above, if added together, are considered as a metavariable measure of health and quality of life. If we take into account the regression analysis, they influence according to their number or frequency and the relationship that social contact has with the elderly person; in fact, living with close relatives is less valued than living with other people. In addition, contacts are influenced not only by how long they last but also by their influence or importance. It is not important to have contact with many different people, but it is important to have contact with those who provide a sense of comfort, who are chosen by the older person, who are not imposed, and, above all, who last long enough to be able to interact, talk, and share common topics of conversation.

As a final summary, we conclude that there is a positive and significant association between the variables described. Therefore, the validity of these scales is confirmed as a proven element for assessing the quality of life in older adults. Multivariable analysis shows that family support or cohabitation is as important for health as social contacts, which are a true welfare factor, and that, by influencing both their number and frequency, they can be considered an estimate of quality of life. Therefore, they are also social factors that generate or predict health in the elderly. At the same time, the deterioration of health in old age requires more participation, more social contacts that act as protectors of health.

Nowadays, due to the higher rate of longevity and the increase in health expenditure, the need to study the psychological, social, and health-related aspects is becoming more and more necessary. The appropriate approach to the aspects mentioned in our study, such as social contact and the main elements of quality of life from a multidimensional perspective, allows us to act as a preventive factor and mitigate the impact of age on health and illness. From this perspective, the psychological and social variables that we have found to be related to the quality of life of older adults are environmental quality, the use of spare time and community facilities, the capacity for personal autonomy and for carrying out basic activities of daily living, and the quantity and quality of social contacts. The combination of all of them makes up a metavariable that acts as a complementary axis to the important medical dimension, which can be understood as an explanatory health factor from an integral, social, and psychic point of view, beyond the absence or presence of illness.

Finally, multidimensional assessment is an effective tool for assessing the quality of life and the objective and subjective health of the elderly. These variables may be related to improving health.

## 5. Conclusions

The biopsychosocial approach is fundamental in the work of professionals dealing with the elderly, as the constant interaction of the different areas of functioning described can explain the causality between them. This is what happens in this case with the relationship between functional skills, leisure, health, and quality of life: when there is greater involvement with others, and more satisfaction in social contacts, there is a better performance of instrumental or daily activities. This can lead to greater autonomy and better control of certain pathologies and physical limitations. However, it is important to mention that the older adult's concept of health, which in this case has been optimal, is a predictor of physical fitness, which is associated with psychological conditions such as life satisfaction, self-esteem, functional skills, activities, participation, and social interaction.

## Figures and Tables

**Table 1:** Internal consistency of the CUBRECAVI tool.

Variable	*N*	Alpha
Health	857	0.61
Social integration	341	0.22
Leisure and activities	862	0.66
Functional capacities	509	0.81
Environmental quality	893	0.73

**Table 2:** Regression analysis of experimental and control groups.

Variable	Environmental group			Control group		
	Beta	*T*	Signification	Beta	*T*	Signification
Health	0.32	5.25	0.001	0.39	2.79	0.001
Functional capacities	0.02	0.11	0.0021	0.19	2.07	0.031
Leisure and activities	0.15	2.89	0.002	0.19	2.09	0.033
Environmental quality	0.9	2.08	0.0026	0.9	0.91	0.02
Income	−0.9	1.45	NS	−0.02	−0.29	NS

Note: variance explained of the experimental group: 25%; variance explained of the control group: 25%; NS = no significance.

**Table 3:** Logistic regression analysis of the association size of the network and social contacts in both groups.

Variable	Environmental group (model 1)				Control group (model 2)			
	*P*	Hazard ratio	95% CI of HR		*P*	Hazard ratio	95% CI of HR	
			Lower limit	Upper limit			Lower limit	Upper limit
*Coexistence*								
Alone (basal)	1				1			
Spouse	0.211	1.068	0.806	1.434	0.325	1.089	0.794	1.387
Son/daughter	0.019	1.289	0.989	1.68	0.389	0.995	0.791	1.335
Siblings	0.003	1.335	1.119	1.988	0.169	1.1	0.833	1.469
Other people	0.008	1.499	1.108	1.995	0.048	1.285	0.886	1.893
*Contacts and weekly visits*								
>5 (basal)	1				1			
2–4 per week	0.499	1.401	0.799	1.132	0.428	0.824	0.659	1.089
1-2 per week	0.599	1.048	0.795	1.244	0.11	1.08	0.88	1.386
0	0.24	1.22	0.78	1.249	0.229	0.795	0.495	1.081
*Times to visit the social network*								
>1-2 day (basal)	1				1			
2–5 per week	0.699	1.024	0.842	1.203	0.504	1.079	0.765	1.109
1-2 per week	0.297	1.097	0.865	1.259	0.069	1.182	0.895	1.34
0	0.1	1.123	0.889	1.295	0.897	0.924	0.828	1.829

Note: model 1 was adjusted by sociodemographic variables in the experimental group; model 2 was adjusted by sociodemographic variables in the control group; 95% CI of HR = confidence interval for *b*.
